# Assessment of long-term quality of life after cavotricuspid isthmus ablation for typical atrial flutter

**DOI:** 10.1186/1477-7525-12-47

**Published:** 2014-04-07

**Authors:** Pilar Cabanas-Grandío, Javier García-Seara, Francisco Gude, José Luis Martínez-Sande, Xesús Alberte Fernández-López, José R González-Juanatey

**Affiliations:** 1 Cardiology Department, University Clinical Hospital of Santiago de Compostela, Choupana, 15706 Santiago de Compostela, Spain; 2 Epidemiology Department, University Clinical Hospital of Santiago de Compostela, Santiago de Compostela, Spain

**Keywords:** Typical atrial flutter, Ablation, Quality of life

## Abstract

**Background:**

Cavotricuspid isthmus (CTI) ablation is the treatment of choice in preventing recurrences of typical atrial flutter (AFl). However, little is known about long-term quality of life (QoL) after CTI ablation.

**Methods and results:**

From 01/2003 to 05/2005, 94 patients who consecutively underwent typical AFl ablation were included in the study. An SF-36 health questionnaire was self-administered before ablation and at follow-up. Transformed scores were calculated, adjusted for age and sex and then normalized and standardized for the Spanish population. Additionally, the minimal important differences (MID) were calculated to assess the smallest change in QoL that patients perceived as positive. A linear regression model was constructed to assess long-term QoL predictors. All SF-36 scales were lower than Spanish population scores. After a mean (SD) follow-up of 6.25 (0.5) years, all scales, except Bodily Pain, were higher than baseline. There was a significant difference for Physical Role (46.4 vs. 38.6, p < 0.001), Vitality (44.4 vs. 41.9, p = 0.038) and Mental Health (46.1 vs. 42.0, p = 0.001). However, only Physical Role achieved the criteria for MID. Recurrence of AFl, basal QoL, history of diabetes mellitus, atrial fibrillation and oral anticoagulation were predictors of long-term QoL.

**Conclusion:**

CTI ablation provides a significant improvement in long-term QoL for the dimensions of Physical Role, Vitality and Mental Health, although the smallest positive change that patients perceive as positive was only observed for Physical Role. Previous diabetes mellitus, atrial fibrillation, oral anticoagulation, basal QoL and AFl recurrence were determinants of a worse long-term QoL.

## Introduction

Typical atrial flutter (AFl) is characterized by a macroreentry circuit around the tricuspid annulus; this circuit contains a propagating wave and an excitable gap. The anterior barrier of the activation wave is formed by the tricuspid annulus, while the posterior barrier is formed by the *crista terminalis*, the eustaquian ridge and its prolongation, the eustaquian valve [1]. The cavotricuspid isthmus (CTI) is a critical component of the circuit; its ablation is the technique available to block the circuit [1-3]. The success rate of this technique is currently high (90 to 100%) and complications are extremely rare. For these reasons, CTI ablation is a first-line treatment [4]. However, there is little information available about the quality of life (QoL) in patients who have undergone typical AFL ablation. There are only a few studies published about QoL; furthermore, in those studies, QoL is assessed using different questionnaires, with short- to medium-term follow-up periods [5]. Our objective was to assess long-term QoL (more than five years) and to evaluate the differences between basal and long-term QoL in patients who had undergone a typical AFl ablation procedure. We did this using the SF-36 questionnaire, which is widely used and validated, and also a new tool for determining the minimal important differences (MID). We also evaluated factors related to long-term QoL.

## Methods

### Study population

A total of 94 patients admitted consecutively to our institution for typical AFl ablation between January 2003 and May 2005 were included in the study.

The inclusion criteria were: a) aged over 18 years, b) the presence of one or more episodes of documented AFl in the previous six months, and c) a demonstration by electrophysiological study of either CTI-dependent AFl or CTI permeability, accompanied by an electrocardiogram taken during a clinical episode consistent with typical AFl in those cases in which the procedure was performed in sinus rhythm.

The exclusion criteria were: a) atypical AFl (non CTI-dependent), b) patients with an implantable cardiac defibrillator (ICD), c) patients who had undergone cardiac surgery or invasive cardiac procedures (coronary angioplasty or cardiac stimulation devices) in the previous month, and d) those patients with a life expectancy of less than one year or patients who were unable to complete the questionnaire.

### Assessment of QoL with the SF-36

All 94 patients included in the study self-administered the SF-36 during the 24–48 hours pre-ablation procedure and at follow-up. The mean (SD) follow-up was 6.26 (0.5) years and was completed for 96.8% of patients.

We use the Spanish version 2.0 of the SF-36 and modified the standard form to include a longer follow-up period.

After all questionnaires had been administered, the 36 items were grouped and coded according to the SF-36 Health Survey manual and its interpretation guide [6,7].

A linear transformation of all scores was performed on a scale from 0 to 100, with 0 representing the worst health status on that scale or dimension, and 100 the best health status.

All dimensions were then standardized and normalized for the Spanish population, adjusted for age and sex, such that the reference values have a mean (SD) of 50 (10). This allowed the results to be compared directly with the reference population, and for scores above or below 50 to indicate better or worse health, respectively, than the average for the Spanish general population. The Physical and Mental Summary Components (PCS and MCS) were also calculated, each of which was also normalized to an overall population mean (SD) of 50 (10).

In addition, to quantify the magnitude of the change in health status, the effect size (ES) and the standardized response mean (SRM) were calculated for each dimension [8,9]. The ES is calculated as the difference between means (before and after an intervention) divided by the SD, prior to the intervention. The SRM is calculated as the difference in the mean values of each dimension between the baseline and follow-up, divided by the SD of the difference between the baseline and the follow-up. Thus, any improvement in positive values reflects the number of times that the result contains the SD of the basal group (ES) or the SD of the difference between the groups (SRM).

The ES and SRM translate the changes into a standard unit of measurement that allows the different measures to be compared: a) <0.20: very small amount of change, b) from 0.20 to 0.5: small amount of change, c) from 0.6 to 0.80: moderate magnitude of change, d) > 0.80: large amount of change.

Another way to quantify the ES is to compare it with the minimal important difference (MID) [9-12]. The MID can be defined as the smallest change in health status that causes a significant change in the patient’s symptoms, justifying the performance or modification of a treatment if there are no significant side effects or excessive costs. It is estimated based on a determination of the standard error of measurement (SEM) and is estimated as 1 x SEM. The SEM is a statistical technique that estimates the possible magnitude of error in a measurement and is defined as being the standard error in an observed result that masks the true result. An important property of the SEM is that its value is independent of the sample. This property means it yields a good estimate of individual changes in a health-related QoL indicator.

$$\mathrm{SEM}={\left(1-r_{d}\right)}^{1/2}\mathrm{in}\;\mathrm{relation}\;\mathrm{to}\;\mathrm{ES}$$

$$\mathrm{SEM}=\left({\mathrm{SD}}_{b}-{\mathrm{SD}}_{d}\right)\;x\;{\left(1-r_{d}\right)}^{1/2}\mathrm{in}\;\mathrm{relation}\;\mathrm{to}\;\mathrm{SRM}$$

r_d_: intraclass correlation coefficient of the differences between baseline and follow-up for each dimension.

SD_b_: baseline SD of each dimension.

SD_d_: SD of the difference between the baseline and follow-up groups.

The ceiling effect and the floor effect were determined for each dimension. The floor value is considered to be the percentage of patients who have the lowest value and the ceiling value is the percentage of patients with the highest value. The floor or ceiling effect is present if more than 15% of patients reach floor or ceiling values within each dimension.

Finally, the long-term QoL predictors were evaluated.

### Statistical analysis

Quantitative variables were expressed in terms of mean and standard deviation if normally distributed, or as within median and interquartile range if the data were clearly skewed. Qualitative variables were expressed as frequencies and percentages.

The statistical Student’s *T* test and the Mann–Whitney *U* test were used to compare the dimensions of the SF-36, according to whether or not normality assumptions were met. A Levene test (homogeneity of variances) and Kolmogorov–Smirnov test (normality assumption) were used to assess the nature of the distribution of each variable. A Wilcoxon rank sum test was used to compare the values of the transformed scores between baseline and follow-up dimensions.

The factors related to long-term QoL (PCS and MCS at follow-up) were determined by using a multiple linear regression model, using the *stepAIC* function (library MASS) and the *lm* function (basic library). The statistical criterion AIC was used, so the lower the value, the better the statistical model. Using this model we identified, based on AIC statistic values, the group which includes all predictors that best explains the dependent variables and that reaches statistical significance, independently of the individual statistical significance of each variable. Both libraries are freely available on the CRAN-R website.

All analyses were performed using SPSS Statistics 17.0 (SPSS Inc., Chicago, IL, USA) and R (CRAN-R, version 2.14.0).

### Ethical considerations

All patients gave written informed consent and this work has the approval of the Clinical Research Ethics Committee of Galicia.

## Results

### Baseline characteristics

Most of the patients were male (82%). The mean (SD) age was 64 (11) years. A total of 44% of patients had a history of atrial fibrillation (AF), 50% received antiarrhythmic drugs (AAD) and 55 (59%) received oral anticoagulation (OAC). The presence of diabetes mellitus (DM) was common (20%), 10 patients (11%) had a history of myocardial infarction, 18 (19%) of heart failure and 21% suffered from Chronic Obstructive Pulmonary Disease (COPD). We documented a component of tachycardiomyopathy in 17 (18%) patients. Table 1 summarizes the baseline characteristics.

**Table 1:** Baseline characteristics

Age (years), mean (SD)	64.4 (10.6)
Men, n (%)	77 (81.9)
Smokers, n (%)	45 (47.9)
Alcohol, n (%)	12 (12.8)
Obesity, n (%)	24 (25.5)
Hypertension, n (%)	46 (48.9)
Diabetes mellitus, n (%)	19 (20.2)
Hyperlipidemia, n (%)	40 (42.6)
Renal failure, n (%)	8 (8.5)
Ischemic heart disease, n (%)	14 (14.9)
Myocardial infarction, n (%)	10 (10.6)
CABG*, n (%)	12 (12.8)
Heart failure, n (%)	18 (19.1)
Dilated cardiomyopathy, n (%)	21 (22.3)
Tachycardiomyopathy, n (%)	17 (18.1)
Valvular heart disease, n (%)	19 (20.2)
- Aortic stenosis	4 (4.3)
- Mitral regurgitation	12 (12.8)
- Tricuspid regurgitation	3 (3.2)
Pacemaker, n (%)	7 (7.4)
Atrial fibrillation, n (%)	41 (43.6)
COPD†, n (%)	20 (21.3)
Asthma, n (%)	5 (5.3)
Steinert, n (%)	2 (2.1)
Arthrosis, n (%)	23 (24.5)
Oral anticoagulation, n (%)	55 (58.5)
Antiarrhythmic drugs, n (%)	47 (50.0)
Betablockers, n (%)	15 (16.0)
Verapamil/diltiazem, n (%)	21 (22.3)
Digoxin, n (%)	12 (12.8)

*CABG: Coronary Artery Bypass Graft. ^†^COPD: Chronic Obstructive Pulmonary Disease.

### Outcomes during follow-up

During follow-up, 16 patients died, three were lost to follow-up and one patient developed a dementia, so was unable to complete the SF-36 questionnaire. Therefore, 74 patients completed the SF-36 at follow-up. The mean (SD) age was 70 (11) years and 82% were male. Hypertension was frequent (68%), 20% suffered from DM and 38% from osteoarthritis. The presence of heart failure and COPD was detected in 12% of patients. During follow-up, 51% of patients developed AF and in 38% of cases the AF was chronic. AFl recurrence was documented in 15 (16%) patients, of whom 14 underwent a new ablation procedure and one patient underwent electrical cardioversion.

### Quality of life outcomes

At baseline, mean scores for all dimensions were lower than mean scores for the Spanish population (mean 50, SD 10). The highest score was obtained for Bodily Pain (Table 2). For Physical Function, PCS and Social Function, scores were approximately six points below the Spanish mean scores. Differences from Spanish mean scores for the other dimensions were more important and the lowest score was for Physical Role (almost 9 points below the Spanish population mean score).

**Table 2:** Differences between follow up and baseline scores standardized and normalized for Spanish population and adjusted by age and sex, at baseline and at follow-up

**Dimension**	**Basal**	**Follow-up**	**Difference**	***P***
Physical function	44.3 (11.9)	44.4 (10.1)	0.56 (9.8)	0.870
Physical role	38.6 (11.7)	46.4 (10.9)	8.93 (13.6)	<0.001
Bodily pain	48.3 (10.7)	44.8 (9.7)	−3.77 (10.6)	0.003
General health	42.8 (9.4)	43.6 (9.9)	1.23 (8.7)	0.226
Vitality	41.9 (10.9)	44.4 (12.0)	2.35 (10.6)	0.038
Social function	43.5 (15.2)	44.2 (13.4)	0.17 (13.6)	0.717
Emotional role	40.4 (15.9)	43.0 (16.0)	2.69 (19.9)	0.142
Mental health	42.0 (11.3)	46.1 (11.4)	4.33 (11.2)	0.001
PCS*	44.6 (10.3)	45.1 (10.1)	0.48 (11.0)	0.596
MCS^†^	41.2 (14.8)	44.5 (15.0)	3.29 (15.4)	0.057

*PCS: Physical Component Summary. ^†^MCS: Mental Component Summary.

At follow-up, mean scores for all dimensions were higher than those obtained at baseline, except for Bodily Pain, but all were lower than the Spanish population mean scores (Table 2).

With respect to the differences between baseline and long term QoL, there was a large difference (greater than eight) for Physical Role (p <0.001), a small difference (between four and six) for Mental Health (p = 0.001) and a very small difference (less than four) for Vitality (p <0.001) and Bodily Pain (p = 0.003); the last of these had the lowest score at follow-up. Table 2 summarizes the scores at baseline and at follow-up, with their differences.

The smallest change in health status perceived by the patient was evaluated using the differences between the QoL at baseline and at follow-up in relation to ES and the SRM. The values were similar for both indicators. Physical Role was the only dimension that achieved the threshold established for MID because the change (0.67 for ES and 0.63 for SRM) exceeded the minimum required to be clinically perceived by the patient on the Physical Role (0.65 for ES and 0.59 for SRM). Table 3 summarizes the values.

**Table 3:** Minimal important differences in relation to effect size and standardized response mean

**Dimension**	**Mean (SD)**	ES*	**SRM†**	**MID (ES)‡**	**MID (SRM)§**
Physical function	0.2 (10.4)	0.08	0.02	0.50	0.58
Physical role	**8.5 (13.4)**	**0.67**	**0.63**	**0.65**	**0.59**
Bodily pain	−3.7 (10.1)	0.32	0.37	0.60	0.63
General health	1.3 (8.8)	0.08	0.15	0.51	0.54
Vitality	2.3 (10.3)	0.23	0.22	0.51	0.53
Social function	0.5 (12.3)	0.05	0.04	0.47	0.58
Emotional role	2.8 (16.3)	0.03	0.17	0.58	0.57
Mental health	4.2 (10.8)	0.35	0.39	0.54	0.56
PCSǁ	0.7 (11.0)	0.05	0.06	0.63	0.60
MCS^#^	3.3 (14.8)	0.22	0.22	0.56	0.57

Physical Role was the only dimension that achieved the threshold established for MID (bold numbers), see text for explanation. *ES: Effect Size. †SRM: Standardized Response Mean. ^‡^MID (ES): Minimal Important Differences in relation to ES. §MID (SRM): Minimal Important Differences in relation to SRM. ǁPCS: Physical Component Summary. ^#^MCS: Mental Component Summary.

At baseline and at follow-up a ceiling effect was determined for Physical Role, Bodily Pain, Social Function and Emotional Role, and a floor effect was determined for Physical Role and Emotional Role, so more questions would probably be required to evaluate these dimensions more effectively in order to assess changes with regard to the baseline scores after typical AFl ablation. Both Role dimensions often have a ceiling or floor effect in the published literature [13,14]. The General Health, Vitality and Mental Health dimensions showed neither a ceiling nor a floor effect, neither at baseline, nor at follow-up.

Factors related to long-term QoL were evaluated using two multiple linear regression models in which the dependent variables were the PCS and MCS, and the independent variables were the baseline PCS and MCS, the history of DM, AF, OAC, heart failure, COPD, osteoarthritis, the occurrence of AF during follow-up and AFl recurrence. The set of variables that best explained the PCS were the baseline PCS, AFl recurrence, previous AF, OAC and DM (p <0.001) (Table 4). For the MCS, the final model included DM and the baseline MCS (p = 0.02) (Table 5).

**Table 4:** Statistical model for the physical component summary

**Variables**	**Estimated coefficient**	Se**	**T value**	***P***
Constant	32.14	4.99	6.4	< 0.0001
DM*	−4.29	2.73	−1.6	0.1202
OAC^†^	−3.91	2.26	−1.7	0.0881
AF^‡^	−3.18	2.25	−1.4	0.1618
Reflutter^§^	−5.91	2.34	−2.5	0.0139
Basal PCSǁ	0.32	0.10	3.1	0.0027
AIC^#^ = 333.43		adjusted R^2^ = 0.244		p = 0.00019

*DM: previous diabetes mellitus. ^†^OAC: previous oral anticoagulation. ^‡^AF: previous atrialfibrillation.§Reflutter: atrial fultter recurrence. ǁBasal PCS: basal Physical Component Summary, adjusted by age and sex. ^#^AIC: model’s statistic. **SE: standard error.

**Table 5:** Statistical model for the mental component summary

**Variables**	**Estimated coefficient**	**Se§**	**T value**	***P***
Constant	38.83	3.21	12.1	< 0.0001
DM*	−5.86	3.02	−1.9	0.0464
Basal MCS^†^	0.18	0.07	2.4	0.0196
AIC^‡^ = 386.61		adjusted R^2^ = 0.077		p = 0.02175

*DM: previous diabetes mellitus. ^†^Basal MCS basal: basal Mental Component Summary, adjusted by age and sex. ^‡^AIC: model’s statistic. ^§^SE: standard error.

## Discussion

The main finding of our study was the improvement of QoL over a long-term period in a group of patients with typical AFl undergoing CTI ablation. This is, to the best of our knowledge, the first study to assess QoL in this population over a long-term period such as this (more than five years) and that used MID to assess QoL changes perceived by the patient. The SF-36 questionnaire is an extensively validated generic QoL measure and has been used in the study of numerous health conditions, including AFl [5]. Unlike other studies, we also used the MID to evaluate differences in QoL. It is important to determine MID because it enables an assessment of whether typical AFl patients perceive changes in QoL produced by CTI ablation as providing enough benefit to make this treatment acceptable, in the absence of excessive cost or severe side effects, regardless of the differences determined by statistical analysis, and thereby an assessment of treatment efficacy. In addition, all scores were standardized and normalized for the Spanish population, adjusted for age and sex, which allowed our results to be compared with the Spanish general population.

Patients had a low score for all SF-36 questionnaire dimensions at baseline. After a long-term follow-up, all dimensions were higher and Physical Role, Vitality and Mental Health significantly improved; however, we only observed a significant MID for Physical Role. This is very important because it indicated that improvements in QoL after CTI ablation are not in fact perceived by the patient in long-term follow-up, except for Physical Role, suggesting that this dimension is particularly affected in AFl and CTI ablation provides an improvement after several years, although, because of its ceiling effect, we cannot know exactly the magnitude of improvement on QoL.

The baseline dimensions were very low and, probably, the existence of comorbidity factors such as DM, heart failure and osteoarthritis partly contributed to these findings. These chronic afflictions have an impact on QoL and, in fact, chronic diseases such as arthritis or osteoarthritis may condition a nine-point difference for the Physical Function dimension, and others, such as DM or heart failure, can explain a difference of up to 13 points [15].

Previous studies that assessed the basal QoL showed similar results [16,17]. The baseline characteristics of our study population are comparable to other studies of AFl ablation [5,16-18]; however, in our population there was a greater proportion of DM (20%, compared to 7–15% described elsewhere). Furthermore, these similar findings were published in relation to other arrhythmias, such as AF [17,19,20], which is usually better tolerated than AFl. Thus, Lönnerholm, et al. [19] reported a reduced basal QoL in patients with AF, both for the Physical and Mental Functions, even when values were lower than those published in the Medical Outcome Study [15], which included patients with symptomatic chronic left ventricular dysfunction (dyspnea, edemas,…), hypertensive patients with very severe symptoms and severe heart failure and/or stroke, patients with previous myocardial infarction and heart failure or patients with severe angina and diabetes with multiorgan involvement.

The basal dimension with the highest score was Bodily Pain, which seems not to be very important role for patients with AFl; indeed, the most common symptoms associated with AFl are palpitations, shortness of breath or occasionally heart failure and, rarely, chest pain [4].

At follow-up, scores were higher than baseline scores for all dimensions of health, except for Bodily Pain, the worst scores for which could be explained by an increase in age-related noncardiac comorbidities such as, for example, osteoarthritis. There was a significant difference between follow-up and baseline scores for Physical Role, Vitality and Mental Health. The improvement for Mental Health and Vitality is important because these dimensions showed neither a ceiling nor a floor effect, which means that the SF-36 questionnaire can reliably perceive positive or negative changes for these dimensions. Moreover, Vitality is an important dimension because it represents the physical and mental aspects of health. However, all scores were low in relation to the Spanish general population. It is possible that associated comorbidities could explain this finding.

Several studies in the literature [16,17,21-23] have described a significant and important improvement in QoL after AFl ablation in short- to medium-term follow-up. In our study, we found a smaller improvement, probably because of the longer follow-up period. Our study extends the follow up and a longer follow-up facilitates the onset of age-related comorbidities and the progression of baseline chronic diseases, such as diabetes mellitus; both phenomena are associated with an impairment on QoL. This fact can contribute to the lower QoL, regardless of the ablation procedure. Even thus, there was an improvement on long-term QoL after CTI ablation with respect to baseline QoL. So, these findings support the AFl conditions an important deterioration on QoL and the ablation provides an improvement on QoL not only in the short- to medium-term, as previously described, but also in the longer term.

The main determinants of long-term QoL that we found were the baseline QoL, AFl recurrence, the previous DM, OAC and AF. AFl recurrence often presents with high ventricular rates, which makes it a very symptomatic arrhythmia; it is more difficult to achieve an appropriate rate control using antiarrhythmic drugs. That is why this arrhythmia has an important impact on QoL. In our study, all patients with typical AFl recurrence underwent a reablation procedure, except for one individual who underwent electrical cardioversion with no more AFl clinical episodes. DM is a chronic progressive disease associated with multiple complications, and so, by the time of follow-up, the disease has progressed and complications will have appeared; this would explain why the deterioration in QoL, both for physical and mental health, was more pronounced in these patients compared to those who do not suffer from DM. It is well known that the presence of AF is associated with impaired QoL, which significantly improves when a good heart rate control or sinus rhythm is achieved. OAC is very likely to be a risk indicator in patients who receive it, indicating that it is used in patients with more comorbidities and therefore at high risk of complications and impaired QoL. In addition, the numerous controls used to optimize the dose of anticoagulant therapy in patients with restricted mobility and who live far from health centers, such as it occurs in our study population, could contribute to a loss of QoL.

So, patients that have undergone AFl ablation constituted a population presenting comorbidities that probably influenced the deterioration of QoL at baseline and at long-term follow-up. Nevertheless, ablation provided a significant improvement, chiefly for Physical Role, Vitality and Mental Health, and patients were able to perceive this improvement for Physical Role.

Finally, the questionnaire completion rate was excellent; all patients answered the questionnaire at baseline (prior to ablation procedure) and at follow-up. Therefore, there were neither blank questionnaires nor incomplete items, unlike other studies [13,24,25] in which the questionnaire was retrospectively completed, making it more difficult to complete and to evaluate the health status before and after a particular treatment.

### Limitations

This study was developed in a single hospital in Spain, so the interpretation of the results should be made with caution. More studies are needed to replicate these findings.

The SF-36 is a generic questionnaire that evaluates most of the health-related areas relevant to multiple diseases and their treatment but, as a generic questionnaire, it is not designed to assess specifically QoL related to the treatment of arrhythmias. Furthermore, there are some important health aspects that are not covered in this questionnaire, such as sleep disorders, eating disorders, familial function, sexual function or cognitive function. In addition, both the ceiling and floor effect were observed for some dimensions, probably indicating that the SF-36 is not quite sensitive and specific enough to detect changes in these dimensions after the ablation procedure.

## Conclusion

CTI ablation of typical AFl provided a significant improvement in Physical Role, Mental Health and Vitality dimensions in a long-term follow-up, but MID was only observed for Physical Role. The main determinants of long-term QoL in these patients were baseline QoL, recurrence of AFl and a history of DM, AF and OAC.

## Abbreviations

AF: Atrial fibrillation; Afl: Atrial flutter; CTI: Cavotricuspid isthmus; DM: Diabetes mellitus; ES: Effect Size; MID: Minimal important differences; OAC: Oral anticoagulation; QoL: Quality of life; SRM: Standardized response mean.

## Competing interests

The authors declare that they have no competing interests.

## Authors’ contributions

PC and JG were responsible for the conceptuation and design of the study, performing electrophysiological procedures, acquisition of data, draft and revision of the manuscript. PC was also involved in acquisition of data, consent inform and Ethical Committee relationships and clinical follow-up. FG: participated in design, statistical analyses and interpretation of data and in manuscript revision, JLM and XF performed electrophysiological procedures and clinical follow-up. Finally JGJ gave the final approval of the version to be published. All authors read and approved the final manuscript.
